# Mitochondrial SLC25 Carriers: Novel Targets for Cancer Therapy

**DOI:** 10.3390/molecules25102417

**Published:** 2020-05-22

**Authors:** Luc Rochette, Alexandre Meloux, Marianne Zeller, Gabriel Malka, Yves Cottin, Catherine Vergely

**Affiliations:** 1 Equipe d’Accueil (EA 7460) Physiopathologie et Epidémiologie Cérébro-Cardiovasculaires (PEC2), Faculté des Sciences de Santé, Université de Bourgogne—Franche Comté, 7 Bd Jeanne d’Arc, 21000 Dijon, France; alexandre.meloux@gmail.com (A.M.); marianne.zeller@u-bourgogne.fr (M.Z.); yves.cottin@chu-dijon.fr (Y.C.); cvergely@u-bourgogne.fr (C.V.); 2 Centre Interface Applications Médicales (CIAM), Université Mohammed VI Polytechnique, Ben-Guerir 43 150, Morocco; gabriel.malka@um6p.ma; 3 Department of cardiology, CHU Dijon Bourgogne, 21000 Dijon, France

**Keywords:** mitochondria, SLC25, protein family, phosphate carrier

## Abstract

The transfer of metabolites through the mitochondrial membranes is a vital process that is highly controlled and regulated by the inner membrane. A variety of metabolites, nucleotides, and cofactors are transported across the inner mitochondrial membrane (IMM) by a superfamily of membrane transporters which are known as the mitochondrial carrier family (MCF) or the solute carrier family 25 (SLC25 protein family). In humans, the MCF has 53 members encoded by nuclear genes. Members of the SLC25 family of transporters, which is the largest group of solute carriers, are also known as mitochondrial carriers (MCs). Because MCs are nuclear-coded proteins, they must be imported into the IMM. When compared with normal cells, the mitochondria of cancer cells exhibit significantly increased transmembrane potentials and a number of their transporters are altered. SLC25 members were identified as potential biomarkers for various cancers. The objective of this review is to summarize what is currently known about the involvement of mitochondrial SLC25 carriers in associated diseases. This review suggests that the SLC25 family could be used for the development of novel points of attack for targeted cancer therapy.

## 1. Introduction

Mitochondria are special cellular organelles that have their own genomic material known as mitochondrial DNA (mtDNA). mtDNA is unique in that it can replicate independently of nuclear DNA. Mutations of almost every mtDNA-encoded gene have been implicated in the production of enhanced reactive oxygen species (ROS), and mtDNA lesions that destabilize bioenergetics and oxidative outputs are a significant cause of human disease [1]. Mitochondria are composed of an inner and outer membrane. Because it is significantly larger, the inner mitochondrial membrane (IMM) must fold upon itself in order to fit within the outer mitochondrial membrane (OMM). The IMM forms invaginations called cristae that extend deeply into the matrix. The cristae define the third mitochondrial compartment, the crista lumen. The crista membranes contain most, if not all, of the assembled complexes of the electron transport chain and adenosine triphosphate (ATP) synthase. The mitochondrial carrier family (MCF) is a group of transport proteins that are most commonly found in the IMM. They facilitate the movement of various solutes across the membrane. The MCF is the largest solute carrier (SLC) subfamily. In 2013, there were 395 known SLCs, and more recent estimates suggest the existence of 456 SLCs (http://slc.bioparadigms.org). SLCs are encoded by the nuclear genome, synthesized by ribosomes, and then imported from the cytosol to their final location [2]. Their principal role is linking the compartmentalized biochemical process between the cytosol and mitochondria. 

## 2. The SLC25 Mitochondrial Inner Membrane Family of Proteins

The SLC25 family (MCF or MCs) transports a great diversity of solutes. All MCF members have a tripartite structure, consisting of three tandemly repeated homologous domains about 100 amino acids in length. The mode of transport varies among different MCs with respect to how the substrate translocation depends on the electrical and pH gradients across the IMM. On the basis of their substrate specificity, MCs can be classified into the following groups: carriers for amino acids, nucleotides and dinucleotides, carboxylates and keto acids, and additional substrates. Each group is further divided into the following subfamilies: citrate (CIC), phosphate (PIC) ADP and ATP (AAC), dicarboxylates (DIC), aspartate, glutamate (AGC), and sulfate (UCPs). The modes of transport can be electroneutral or electrogenic [3,4,5].

### 2.1. Characteristics of SLC25

Members of the MCF link cytosolic and mitochondrial metabolism by facilitating transport across the IMM. Whereas the OMM is relatively permeable to solutes up to 5 kDa due the presence of voltage-dependent anion channels, the IMM is comparatively impermeable, allowing it to maintain efficient oxidative phosphorylation. The transported substrates include nucleotides, amino acids, cofactors, carboxylic acids, and inorganic anions. Most mitochondrial transporters operate according to a strict exchange mechanism [3].

#### 2.1.1. Role of Calcium and Magnesium in Relation to Mitochondrial SLC25 Proteins

Members of the SLC25 family include Ca^2+^-dependent mitochondrial carriers (CaMCs): LCaMCs (long CaMCs) and SCaMCs (short CaMCs). LCaMCs are the mitochondrial aspartate-glutamate transporters. SCaMCs (also called APCs) are the mitochondrial ATP-Mg^2+^/Pi transporters, and ATP-Mg^2+^/Pi and aspartate/glutamate transport activities are modulated by various signals such as calcium [5,6].

**Figure 1:**
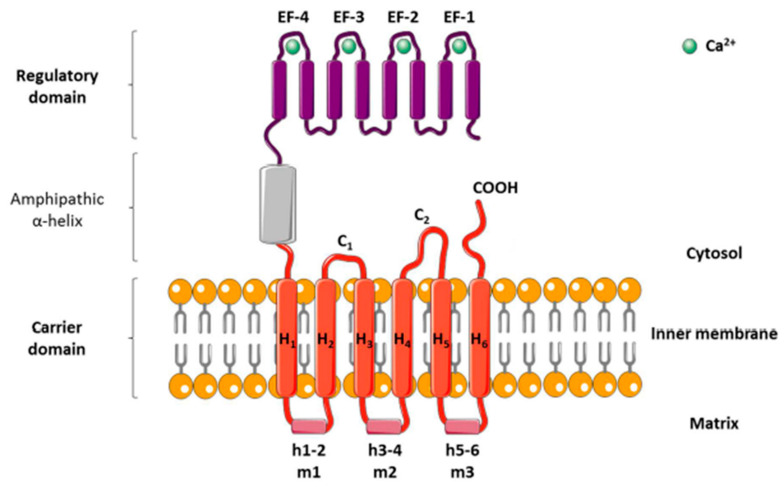
Structural model of the ADP/ATP carrier (AAC). The carrier consists of three separate domains: (1) an *N*-terminal regulatory domain with four calcium-binding EF-hands, (2) a loop domain containing an amphipathic α-helix, and (3) a mitochondrial ADP/ATP carrier. The carrier domain has six transmembrane helices (H1 to H6); three matrix loops (m1, m2, and m3), each containing a small hydrophilic helix (h1–2, h3–4, and h5–6); and two cytosolic loops (c1 and c2).

In humans, genes encode full-length APC paralogs; SLC25A24, SLC25A25, SLC25A23 and SLC25A54 encode for SCaMC isoform 1 (APC-1), SCaMC-2 (APC-3), SCaMC-3 (APC-2), and SCaMC-1L, respectively [7,8]. APC has a three-domain structure. The N-terminal domain of APC forms a calcium-sensitive regulatory domain, and the presence of four EF-hands has been demonstrated. The N-terminal regulatory domain shares sequence identity with four EF-hand containing proteins. The C-terminal carrier domain consists of six transmembrane alpha-helices and three short matrix alpha-helices. When calcium binds to the regulatory domain of APC, there is a stimulation of the substrate transport activity of the carrier (Figure 1).

#### 2.1.2. Metabolic Role of Mitochondrial SLC25 Proteins

##### Mitochondrial Phosphate Carrier (PiC)

A deficiency of the mitochondrial phosphate carrier SLC25A3 has been proven to lead to a disorder in the synthesis of ATP. The importance of SLC25A3-encoded proteins in facilitating energy production is highlighted by the profound disease phenotype observed in patients presenting with mutations in the skeletal muscle-specific isoform of this gene. Affected individuals present with various disorders [9]. In the MCF, two PiC isoforms, i.e., PiC A and PiC B, transport Pi to the mitochondrial matrix to supply the Pi that is required for ADP phosphorylation. H_2_PO_4_^−^ (shown as Pi), H^+^, Ca^2+^, Mg^2+^, and adenine nucleotides cross the OMM via voltage-dependent anion channel (VDAC). The PiC mediates electroneutral cotransport of Pi and H^+^ across the IMM. Pi is used for phosphorylation of ADP (entering the matrix through adenine nucleotide translocase (ANT) by the ATP synthase).

PiC encoded by the nuclear gene SLC25A3, catalyzes the transport of inorganic phosphate (Pi) into the mitochondrial matrix; transport is electroneutral and either in symport with H^+^ or in exchange for OH^−^. The following two main alternative transcripts of the human SLC25A3 gene (chromosome 12q23) have been described: the “standard” SLC25A3 transcript variant 1 and the SLC25A3 transcript variant 2. The corresponding protein products are isoform A and isoform B, respectively. The differential isoform expression in various organs suggests the presence of regulatory regions responding to tissue specificity in the SLC25A3 genes [10,11]. Several members of the SLC25 group have been reported to play roles in glucose-stimulated insulin secretion (GSIS) in pancreatic β-cells. In these cells, research has demonstrated the critical role of Pi influx to mitochondria in ATP production through the SLC25A3 carrier. In pancreatic β-cells, the ATP increase is slight, and the ADP decrease is prominent via an increase in glucose levels beyond the triggering level of insulin secretion [6].

Phosphate levels can control copper availability or transport. SLC25A3 acts as a phosphate and copper transporter, and the depletion or deletion of SLC25A3 decreases the total mitochondrial copper levels. The mechanisms by which metals are imported into mammalian mitochondria are generally poorly defined. Finally, the mammalian phosphate carrier SLC25A3 is a mitochondrial copper transporter that is required for cytochrome c oxidase biogenesis [12].

##### SLC25A23/ SLC25A24 /SLC25A25 and Homeostasis

The SLC25 family contains Ca^2+^-dependent mitochondrial carriers (CaMCs). The flow of Ca^2+^ across the IMM regulates cellular bioenergetics, intracellular cytoplasmic Ca^2+^ signals, and various cell death pathways. Ca^2+^ uptake into the mitochondria requires active anions such as Pi, acetate, or bicarbonate that can provide a source of H^+^. Pi facilitates the uptake of Ca^2+^ and the mitochondrial calcium uniporter (MCU) [13]. ATP is required to maintain mitochondrial ion homeostasis, which is essential for MCU-mediated Ca^2+^ uptake. The ATP-Mg solute carriers (SLC25A23, SLC25A24, and SLC25A25) transport adenine nucleotides to the matrix of the mitochondria in response to cytosolic Ca^2+^ [14]. Mitochondrial Ca^2+^ plays a key role in glutamate excitotoxicity, and the prevention of Ca^2+^ uptake by mitochondria protects against neuronal death. Glutamate is an excitatory neurotransmitter, and Ca^2+^ overload plays a major role in excitotoxicity [15].

SLC25A24 is responsible for the transport of nucleotides, metabolites, and cofactors across the IMM. SLC25A25 is thought to control ATP homeostasis by functioning as a Ca^2+^-regulated shuttle of ATP-Mg/Pi. Mice with an inactivated *Slc25a25* gene were found to have reduced metabolic efficiency as evidenced by enhanced resistance to diet-induced obesity and impaired exercise performance [16]. Studies on the function of SLC25A23 found that a deficiency in this protein increased neuronal vulnerability to excitotoxicity both in vitro and in vivo, and it was thought that SLC25A23 was able to maintain mitochondrial ATP levels [17]. SLC25A23 appears to be a promising target in strategies aimed at increasing resistance to excitotoxicity in the brain.

Recurrent mutations in SLC25A24 are responsible for the Fontaine syndrome, which is a human progeroid syndrome characterized by prenatal and postnatal growth retardation, and craniosynostosis syndromes [18].

### 2.2. Cellular Iron and Mitochondrial SLC25 Proteins

Iron is essential for mitochondrial function, and mitochondria are intimately involved in the regulation of cellular iron. Iron plays a role in the generation of ROS both directly and indirectly via the Fenton and Haber–Weiss reactions. An elaborate system has evolved, making it possible to stringently regulate the concentrations of free iron and oxygen in various sites of the body [19]. Among the regulators, there appear to be two essential transporters, i.e., SLC25A28 and SLC25A37 [20]. The transport of iron into the mitochondrial matrix is required for the formation of iron–sulfur clusters, which are involved in a number of mitochondrial enzymes and metabolic processes [21,22].

## 3. Mitochondria and the Transport of Substrates

### 3.1. Mitochondrial Bioenergetics and Metabolic Functions

Adenine nucleotide translocase (ANT) mediates the exchange of ATP/ADP between the mitochondrial matrix and the intermembrane space (IMS). Under physiological conditions, voltage-dependent anion channels (VDACs) and ANT are functionally coupled, which allows for the efficient transfer of metabolites, and VDAC1 and ANT are found to have direct structural interactions. VDACs exist in the OMM of all eukaryotic organisms. VDAC channels can exist in a variety of structural states, exhibiting a range of selectivity. As a membrane channel, its fundamental function is to facilitate and regulate the flow of metabolites between the cytosol and the mitochondrial intermembrane space. It has been reported that VDAC has a dual role in mitochondrial functions, in association with PiC. [23]. VDACs exist as the following three isoforms in mammals: VDAC1, VDAC2, and VDAC3. Among the three isoforms, VDAC1 is the most abundant in heart mitochondria and in the OMM. Along with oxidative stress, it plays a role in the modulation of cardiac injury such as ischemia/reperfusion [24]. VDAC controls the diffusion of superoxide anion (O_2_^•−^) from the IMS to the cytosol, and VDAC gating has a voltage-dependent profile and displays significant selection for anions [20].

**Figure 2:**
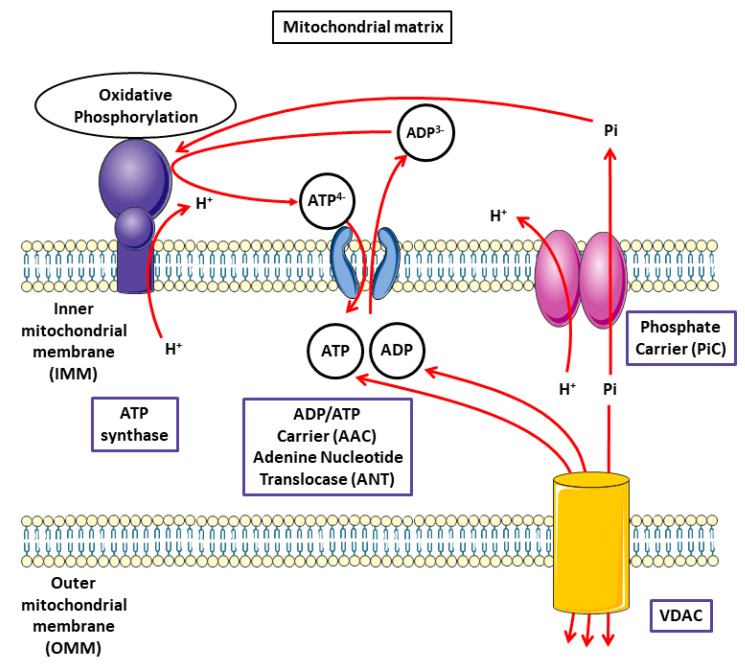
Schematic representation of the functions of carriers regulating the adenosine nucleotide pools. The ADP/ATP transport is characterized as an exchange of exogenous and endogenous nucleotides. ADP/ATP exchange across the inner mitochondrial membrane is catalyzed by the ADP/ATP carrier (AAC). The phosphate carrier protein and the voltage-dependent anion selective channel (VDAC) are transmembrane proteins located in the mitochondrial membrane catalyzing the transport of Pi. The process of oxidative phosphorylation permits the generatation of ATP from ADP, and Pi drives ATP synthesis by ATP synthase through H^+^ reuptake.

In most cell types, the mitochondria form a dynamic network that is continuously remodeled by fusion and fission of the organelles. A disruption in the mitochondrial bioenergetics and metabolic function can lead to a number of disorders [25]. Mitochondria and the nucleus have a complex relationship with both anterograde (nucleus to mitochondria) and retrograde (mitochondria to nucleus) signaling, and the most prominent contribution of mitochondria to cellular metabolism is their ability to generate ATP through the tricarboxylic acid (TCA) cycle and the mitochondrial oxidative phosphorylation (OXPHOS) process [26]. ATP is the energy carrier compound that is mainly produced in chloroplasts and mitochondria. In both organelles, its production results from oxidation-reduction reactions performed by multienzymatic complexes located in lipid-bilayer membranes. These reactions are coupled to a proton gradient which is used by the ATP synthase to synthesize ATP from ADP and inorganic phosphate (Figure 2).

### 3.2. Mitochondrial Oxidative Phosphorylation (OXPHOS) Process

The cellular energy production that is achieved in the mitochondria through OXPHOS is especially important for organs with high demands in energy such as the heart. The OXPHOS process uses ATP synthase and four multienzymatic respiratory complexes (complexes I–IV) which are embedded in the inner mitochondrial membrane. NADH and succinate produced by the Krebs cycle are oxidized by complex I (NADH, ubiquinone oxidoreductase) and complex II (succinate, ubiquinone oxidoreductase), respectively, and the electrons are transferred to the ubiquinone pool, leading to the reduction of ubiquinone to ubiquinol within the mitochondrial membrane. The respiratory chain transforms O_2_ into H_2_O in the mitochondrial matrix, but O_2_^•−^ is also generated. It has been calculated that less than 0.1% of the electrons passing through the respiratory chain leak onto O_2_ to form superoxide in normal conditions of electron transfer. ROS, including O_2_^•−^, hydrogen peroxide (H_2_O_2_), and their derived forms such as hydroxyl radical (HO∙) are implicated in various cell-signaling processes.

O_2_^•−^ will also interact nonenzymatically with nitric oxide (NO^•^) to produce peroxynitrite (ONOO^−^) to form reactive nitrogen species (RNS). NO^•^ production comes from a variety of nitric oxide synthase (NOS) complexes including endothelial NOS (NOS3), neuronal NOS (NOS1), inducible NOS (NOS2), and possibly a mitochondrial NOS (mtNOS). Since NO^•^, a radical gas, is largely membrane permeable, its production at any cellular location can be assumed to have free access to the mitochondrial area. Ca^2+^ is required to activate NOS3 and NOS1, which can, then, further increase mitochondrial NOS and ROS production. In these spatial and temporal conditions, redox signaling is recognized for its role in mediating various tissue-specific cellular functions and diseases [27,28]. In this field, some disorders including functions in the neuronal, immune, and cardiovascular systems were associated with the overproduction of ROS and RNS [24]. In physiological conditions, OXPHOS homeostasis is tightly controlled by the mitochondrial transporters that are located in IMM and OMM. An appropriate balance between ATP production and ROS functions is essential for maintaining cellular homeostasis, and the role of mitochondria uncoupling proteins (UCPs) is key to maintaining stability. The main function of UCP1 is to regulate the flux of protons through the ATP synthase. The ubiquitous UCP2 is associated with several conditions that include cardiovascular diseases, metabolic diseases, and cancer [29].

### 3.3. Characterization of Pi Transport in Mitochondria: Role of the PiC and Adenine Nucleotide Translocase (ANT) in Oxidative Phosphorylation

As we reported previously, Pi is used for the phosphorylation of ADP by ATP synthase; PiC transports Pi to the mitochondrial matrix, passing through the OMM via VDAC.

PiC, referred to as SLC25A3, has been cloned from bovine and human heart tissue. Human SLC25A3 was located at chromosome 12q23 encoded by slc25a3 genes. PiC protein consisted of 362 (isoform A) or 361 (isoform B) amino acids [30].

The role of the PiC in ATP production and OXPHOS has been confirmed in mammals using mouse models of PiC depletion [31] or by studying a loss-of-function mutation in the human SLC25A3 gene [32]. After mutation of human SLC5A3 gene, functional investigation of intact mitochondria showed a deficiency of ATP synthesis in muscle but not in fibroblasts, which correlated with the tissue specific expression of exon 3A in muscle versus exon 3B in synthesis.

The properties of ANT have been thoroughly explored in research. It is a protein abundantly found on the IMM and primarily involved in ADP/ATP exchange. Passing a large substrate through a small protein requires significant conformation changes [4]. Human ANTs exist in four isoforms (ANT1-4) encoded by different genes as follows: SLC25A4, SLC35A5, SLC25A6, and SLC25A31. ANT1 is mainly expressed in muscle and brain tissue, ANT2 is expressed in proliferating tissue, ANT3 has a ubiquitous pattern of expression, and ANT4 is localized to the testes [33]. ANT plays a central role in the regulation of the rate of ATP production demonstrated in mitochondria isolated from human skeletal muscle [34].

**Scheme 1:**
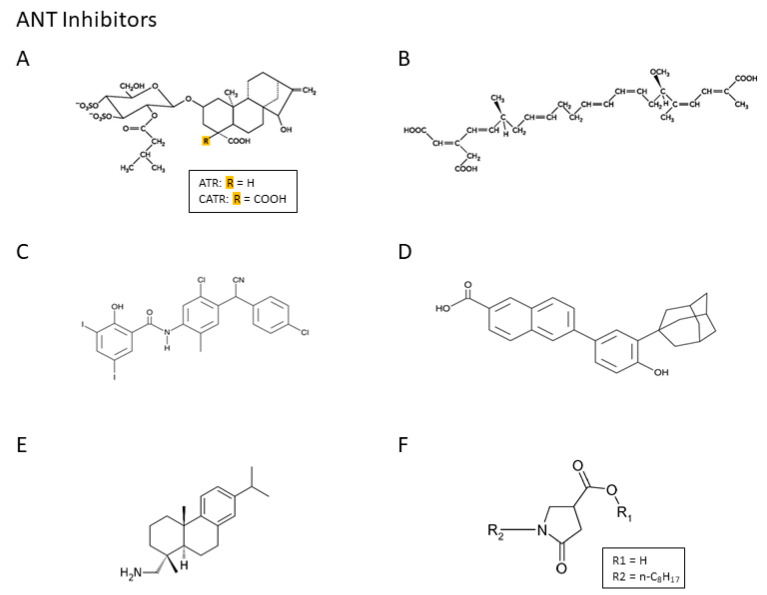
Adenine nucleotide translocase (ANT) inhibitors. (**A**) Atractyloside (ATR) and carboxyatractyloside (CATR) are two natural heteroglucosides that are produced in some plants such as *Atractylis gummifera*. ATR is a hydrophilic glycoside. A modified variant of an atractylenolide carries an additional carboxyl group at the C4 atom of the atractyligenin, which is then referred to as CATR; (**B**) Bongkrekic acid (BA) is a natural poison secreted by the bacteria *Pseudomonas cocovenenans* and the Gram-negative aerobic bacteria Cocovenenans. BA is a polyunsaturated long-chain fatty acid derivative (heptaenedioic acid derivate); (**C**) Closantel is a salicylanilide compound with narrow-spectrum anthelmintic action; (**D**) CD437 is the prototypical adamantyl arotinoid of the retinoid-related molecule family; (**E**) Leelamin is a naturally occurring product derived from the bark of pine trees. It is a diterpene molecule whose name derives from the Sanskrit word leela which means “play”. It has weak affinity for the human central cannabinoid (CB1) and peripheral cannabinoid (CB2) receptor; (**F**), MT-21 is an N-alkylpyrrolinone derivate.

The use of ANT inhibitors has provided a detailed image of the precise role of ANT in cellular functions, and the pharmacologic inhibition of ANT and ATP synthases have shown promise as potential molecular targets for human diseases. Atractyloside (ATR), carboxyatrac-tyloside (CATR), and bongkrekic acid (BKA), which are naturally occurring substances, have been extensively studied as ANT inhibitors, particularly because of their high specificity for ANT. They inhibit translocase activity in a concentration-, isoform-, and expression system-dependent manner. Closantel, CD437, and leelamine are modulators of ANT function (Scheme 1), but differences in the level of expression of the ANT isoforms can affect the modulatory activity of these compounds. Currently, in clinical practice, these agents could prolong overall survival but with a lot of adverse events. In the literature, it has been suggested that acyl-CoA had an inhibitory role on ADP/ATP transport in a starved liver, but acyl-CoA was not found to produce a physiological regulation of ADP/ATP transport [35].

In pathological situations such as ischemia/reperfusion, ATR or BKA binding on the ANT inhibitor has reduced the interaction of ANT and VDAC in vitro. It is not known if oxidant stress conditions (such as excess ONOO^−^ production from the reaction of O_2_^•−^ and nitric oxide (NO^•^) during ischemia/reperfusion injury) induces a tyrosine nitration-driven modification of ANT or whether it alters the interaction between ANT and VDAC [36]. Concerning the relationship between the properties of ANT and the oxidative process, it has been demonstrated that mitochondrial exposure to exogenous peroxynitrite leads to tyrosine nitration of ANT, which is associated with decreased mitochondrial bioenergetics. ONOO^−^-mediated mitochondrial cytochrome c (cyt c) release can be attributed to increased permeability of the mitochondrial membranes. More specifically, the release of cyt c from mitochondria that is induced by ONOO^−^ treatment has been attenuated by pretreatment with either a VDAC inhibitor or an ANT inhibitor [37]. Given the importance of ANT to mitochondrial physiology, mutations or altered expression have been found to be associated with many human diseases [38].

In addition to its role in energy metabolism, PiC has been implicated in regulating cell death as a modulator of mitochondrial permeability transition pores (MPTP) in the IMM [39,40]. The MPTP is a nonselective pore that can pass both ionic and nonionic substrates. Elevated Ca^2+^ is involved in the formation of MPTP, which is followed by increased membrane permeability. This process results in the modification of membrane potential, mitochondrial swelling, and ultimately cell death [40]. The importance of PiC in facilitating energy production is demonstrated by the severe disease phenotype observed in individuals with genetic mutations in the skeletal-muscle-specific isoform gene. Mitochondrial-driven hypertrophic cardiomyopathy has been observed in both the mouse models of Slc25a3 deletion and in humans with phosphate carrier deficiency [31,41].

### 3.4. Role of Gasotransmitters on Mitochondrial Carriers

Interestingly, gasotransmitters, such as NO, carbon oxide (CO), and hydrogen sulfide (H_2_S) are not only endogenously produced but also exert beneficial effects such as anti-inflammation and cytoprotection at low concentrations. These gasotransmitters specifically target the mitochondria [42]. Some mechanisms, including antioxidant action, the regulation of ion channels, and preservation of mitochondrial functions, could be responsible for the protective effect of H_2_S on various structures [43]. Two members of SLC25, BMCP1 (brain mitochondrial carrier protein 1, encoded by SLC25A14) and KMCP1 (kidney mitochondrial carrier protein 1, encoded by SLC25A30), catalyze the transport of anions and thiosulfate, which are produced by H_2_S degradation in the mitochondria. They play a role in the modulation of H_2_S levels [44]. CO was able to uncouple mitochondrial respiration when delivered to isolated rat heart mitochondria using a water-soluble CO-releasing molecule (CORM-3) [45]. CORM-3 activates PiC, leading to an increase in phosphate and proton transport inside mitochondria, inducing an uncoupling effect [46]. These results demonstrated that CO activated PiC, which led to an increase in intra-mitochondrial phosphate and protons, potentially participating in the uncoupling effect induced by CO.

### 3.5. Role of ATP-Mg/Pi Carrier in Cellular Functions and Disease

#### 3.5.1. Overview of the Regulation of ATP-Mg/Pi Carrier

Phosphate is an abundant molecule that is incorporated into ATP and transferred from ATP to a large number of small biomolecules including nucleotides, sugars and lipids, and also macromolecules such as proteins.

Mitochondrial PiC transports Pi to the mitochondrial matrix to supply the orthophosphate (Pi) required for ADP phosphorylation. PiC exists as two isoforms (PiC A and PiC B) as a result of alternative splicing events that differ in the presence of two separate exons (3A and 3B) [47].

The uptake of orthophosphate (Pi) into the mitochondrial matrix is essential for the oxidative phosphorylation of ADP to ATP. The mitochondrial phosphate transporter/carrier (MPT/PiC), which is located in the IMM, catalyzes the phosphate (H_2_PO_4_)/proton symport, the phosphate/hydroxyl ion antiport, and the exchange of the mitochondrial matrix with cytosolic phosphate. Isoform A is found abundantly in the heart, skeletal muscle, and diaphragm tissues to match the higher energy demands, whereas isoform B is present in all tissues to provide basic energy requirements.

Pi uptake can also be mediated by other carriers such as ATP-Mg/Pi. The transport properties and mitochondrial targeting of these carriers indicate that they are isoforms of the ATP-Mg/Pi carrier described in whole mitochondria [48]. ATP-Mg/Pi carrier activity is known to be regulated by Ca^2+^ in isolated mitochondria and by Ca^2+^-mobilizing hormones.

In addition, ATP is rapidly synthetized from ADP and phosphate (Pi) by ATP synthase. Newly synthetized ATP in the mitochondrial matrix is exchanged for cytosolic ADP by the mitochondrial AAC. This carrier, which is named adenine nucleotide carrier (Ancp or ANC), is a nuclear encoded protein that catalyzes the exchange of ATP^4-^ (generated in the mitochondria by ATP synthase) with ADP^3-^ (produced in the cytosol by most energy-consuming reactions). Human AACs are involved in different genetic diseases and play a role in cancerogenesis. The following two specific inhibitors have greatly facilitated biochemical and biophysical studies of the carrier: carboxyatractyloside (CATR), which is known to inhibit respiration, and BKA [49].

Mitochondria have ATP-Mg/Pi carriers (APC), which transport ATP, with or without magnesium and ADP in exchange for matrix Pi. This unbalanced exchange can rapidly alter the concentration of adenosine nucleotide in the matrix, which is fundamental for cellular growth and energy metabolism. The ATP-Mg/Pi carrier is saturable and activated by calcium [48]. APC has the important role of altering the mitochondrial adenine nucleotide pool in order meet the fluctuating energy demands within the cell [8].

Defects in human ANC isoforms can arise from transcriptional or translational deregulation or protein inactivation. A shortage of mitochondrial phosphate carriers can cause severe neonatal lactic acidosis, hypertrophic cardiomyopathy, and generalized muscular hypotonia [9]. Autoantibodies against ANC1 have been described in patients suffering from dilated cardiomyopathy (DCM), a cardiac defect affecting both ventricles and septum [50]. ANC dysfunction can affect different tissues at various levels, and for this reason the symptoms can vary from one patient to another.

#### 3.5.2. SLC25 Family and Cancer

A direct link between abnormal SLC25 activity and disease has been demonstrated in cases of cancer. Concerning CIC, it has been found to be involved in inflammation and cancer [5,33]. SLC25 members were identified as potential biomarkers for various cancers. In this context, SLC25 carriers are potentially exploitable targets for anticancer strategies.

SLC25A1 is overexpressed in most lung cancers relative to normal tissues and in metastatic sites. It has been demonstrated that SLC25A1 plays a key role in the adaptive mechanisms that allow some tumor cells to acquire drug resistance. Studies have shown that SLC25A1 is an essential component of the tumor cell metabolism and have enlightened novel mechanisms and therapeutic perspectives for the treatment of resistant non-small cell lung cancer tumors [51]. CIC appears as an important determinant of the homeostatic control of tumor mitochondria, through which activity CIC becomes essential to the cancer promoting metabolic program. It has been reported in tumors that high CIC levels preserved a critical threshold of mitochondrial activity and amount that allows adaptation during metabolic and respiration stress. In this context, it appears rational to test the effects CIC inhibitors in human pathogenic conditions such as cancer [52]. It has been reported that the frequency of p53 mutations was particularly high in lung, ovarian, and breast cancers, paralleling high expression levels of CIC in these tumors. In a therapeutic approach, CIC is an important target for cancer therapy; CIC promotes tumorigenesis while its inhibition reduces tumor growth [53].

Concerning SLC25 A10, its major role is to transport the dicarboxylate substrates (malate and succinate) out of the mitochondria in exchange for phosphate, sulfate, and thiosulfate. The substrates of the SLC25A10 carrier are linked to NADPH synthesis and the regulation of cell metabolism, and they are involved in the regulation of redox homeostasis [54]. Specifically, the SLC25A10 carrier helps to regulate redox homeostasis in order to protect confluent cells against oxidative stress. Interestingly, increased SLC25A10 expression has been seen in a variety of tumor types. A549 cell lines with downregulated SLC25A10 have an altered growth process and a decreased ability to respond to oxidative stress [54]. Changes in energy metabolism and redox homeostasis are frequently identified in tumor cells, which are able to adjust their metabolism phenotypes to adapt to the microenvironment [55]. The change in redox homeostasis has been linked to a shift in metabolic energy, going from glycolysis to mitochondrial oxidative phosphorylation [56].

Many recent studies have confirmed that the circadian clock plays a key role in the daily metabolism of vital organs. In addition, the internal medium of a cell changes according to a ~24 h cycle that is regulated by a molecular clock [57]. In mammals, the circadian rhythm is mainly maintained by three linked transcriptional feedback loops that contain transcriptional regulators. One of these regulators is the activator known as circadian locomotor output cycles kaput (CLOCK) [58]. It has been recently reported that SLC25A10 is a site that responds to CLOCK binding. Rhythmic interactions between CLOCK and SLC25A10 lead to the circadian regulation of SLC25A10 and mitochondrial metabolism [59]. The nuclear receptors REV-ERBα and REV-ERBβ are involved in the cell-autonomous circadian transcriptional/translational feedback loops and act as transcriptional repressors. Molecules acting on REV-ERBs have been used to elucidate the connections between circadian rhythm and breast cancer such as human epidermal growth factor receptor 2 HER2+ subtype [60,61]. The circadian nature of mitochondrial morphology through fusion/fission mechanisms, and its relation to metabolic rhythm and clock regulation have been thoroughly studied in the literature [62].

Two MCs for aspartate and glutamate, referred to as AGC1 (SLC25A12 or Aralar1) and AGC2 (SLC25A13 or citrine), have been identified in humans [63]. The mitochondrial AGCs are important to supply aspartate to the cytosol with potential therapeutic value in carcinoma. In humans there are two AGC isoforms, i.e., AGC1 and AGC2 encoded by SLC25A12 and SLC25A13, respectively. SLC25A12 upregulation in hepatocellular carcinoma (HCC) cell lines is essential to promote HCC cell growth. The SLC25A12 gene, expressed in adult liver, is upregulated in HCC cell lines by epigenetic mechanisms and AGC1 is involved in HCC cell growth and migration by supplying cytosolic aspartate levels for nucleotide biosynthesis. In this field, aspartate has been described as a limiting metabolite for cancer growth [64,65].

Among the other transporters known as MCF were investigated the properties of the following two human UCPs: UCP5 (BMCP1, brain mitochondrial carrier protein 1 encoded by SLC25A14) and UCP6 (KMCP1, kidney mitochondrial carrier protein 1 encoded by SLC25A30). UCP5 and UCP6 transport inorganic anions such as sulfate and thiosulfate. It has been demonstrated that UP5 overexpression lowers the accumulation of ROS in neuronal and neuroblastoma cells [66].

Research has found that the gene for the SLC25A43 mitochondrial transporter is commonly deleted in HER2+ breast cancer, as well as in other cancers, and altered SLC25A33 expression influences the proliferation of breast cancer cells [67]. Finally, it appears that cancer cells that alter mitochondrial function are able to modify energy metabolism to sustain uncontrolled proliferation.

When compared with normal cells, the mitochondria of cancer cells exhibit significantly increased transmembrane potentials and a number of their transporters are altered. These differences have been used as the basis for developing mitochondria-targeting compounds, such as triphenylphosphonium (TPP), which may be preferentially accumulated within the mitochondria of tumor cells [68]. Doxorubicin, an anticancer drug that intercalates into DNA and inhibits tropoisomerase II [69], has been conjugated with a selective mitochondria-localizing compound; the new agent enhances its selectivity towards cancer cells, acting on the properties of the mitochondrial transporters.

Inhibiting the expression of a carrier such as SLC25A10 can make it possible to reprogram cell metabolism, compromise cell growth, and increase sensitivity to traditional anticancer drugs. For instance, pharmacologic inhibition using butylmalonate (BMA) in combination with ionizing radiation was able to overcome the increased radioresistance induced by adaptation to chronic-cycling hypoxia [70]. Pharmacologic inhibition of SLC25A10 has been proven to be a novel therapeutic strategy to counteract increased radioresistance induced by chronic-cycling hypoxia. It is an important topic because there is a high demand in the development of novel therapeutic advances.

On the basis of the evidence of altered expression of SLC25A10, it has been suggested as a novel target for anticancer strategies. Confluent non-small cell lung cancer (NSCLC) cell line A549 with downregulated SLC25A10 has altered growth behavior and decreased ability to respond to oxidative stress, especially in resting cells. This study confirms that SLC25A10 has an important role in regulating redox homeostasis. The inhibition of SLC25A10 expression is a potential strategy to reprogram cell metabolism, compromise cell growth, and increase sensitivity to anticancer drugs such as cisplatin [54].

Members of the SLC25 family can be involved either directly or indirectly in physiological and pathological processes. Studies have demonstrated that PiC has a potential role in mitochondria-dependent cell death; the overexpression of this carrier triggers the intrinsic apoptosis pathway [33,71].

Recent results in type A549 cells suggest a link between metabolic alterations and p21 expression with cumulative effects of decreased SLC25A10 expression and metformin treatment. SLC25A10 knockdown and metformin treatment in A549 cells worked together to influence metabolism and mitochondrial ROS production. Interestingly, metformin treatment decreased the expression of the SLC25A10 carrier, suggesting that this drug could inhibit tumor growth when used in cancer treatment [72].

## 4. Conclusions

In conclusion, the SLC25 family of transporters transports a vast range of solutes, and the members of this family were identified as potential biomarkers for various cancers. Inhibition of the expression of carriers such as SLC25A10 can be used as a strategy to reprogram cell metabolism, compromise cell growth, and increase sensitivity to traditional anticancer drugs. An important outcome of the molecular identification and characterization of SLC25 transporters has been the discovery of several SLC25-related diseases. Another subject that warrants further research is the possible implication of polymorphic SLC25 genes in certain disorders [20]. Future experiments should focus on treatment strategies for SLC25-derived diseases. In the future, successful mitochondria-targeting agents will need to possess a variety of clinically relevant characteristics, including organ selectivity and durable accumulation in the mitochondria.
